# Building integral projection models: a user's guide

**DOI:** 10.1111/1365-2656.12178

**Published:** 2014-01-20

**Authors:** Mark Rees, Dylan Z Childs, Stephen P Ellner, Tim Coulson

**Affiliations:** 1 Department of Animal and Plant Sciences, University of Sheffield, Western BankSheffield, S10 2TN, UK; 2 Department of Ecology and Evolutionary Biology, Cornell UniversityIthaca, NY, 14853-2701, USA

**Keywords:** integral projection model, mathematical model, Soay Sheep, structured population

## Abstract

1. In order to understand how changes in individual performance (growth, survival or reproduction) influence population dynamics and evolution, ecologists are increasingly using parameterized mathematical models.
2. For continuously structured populations, where some continuous measure of individual state influences growth, survival or reproduction, integral projection models (IPMs) are commonly used.
3. We provide a detailed description of the steps involved in constructing an IPM, explaining how to: (i) translate your study system into an IPM; (ii) implement your IPM; and (iii) diagnose potential problems with your IPM. We emphasize how the study organism's life cycle, and the timing of censuses, together determine the structure of the IPM kernel and important aspects of the statistical analysis used to parameterize an IPM using data on marked individuals.
4. An IPM based on population studies of Soay sheep is used to illustrate the complete process of constructing, implementing and evaluating an IPM fitted to sample data.
5. We then look at very general approaches to parameterizing an IPM, using a wide range of statistical techniques (e.g. maximum likelihood methods, generalized additive models, nonparametric kernel density estimators). Methods for selecting models for parameterizing IPMs are briefly discussed.
6. We conclude with key recommendations and a brief overview of applications that extend the basic model. The online Supporting Information provides commented R code for all our analyses.

## Introduction

The development of data-driven, study-specific models is now commonplace in population biology (Ozgul *et al*. 2010; Bruno *et al*. 2011; Childs *et al*. 2011; Wallace, Leslie & Coulson 2013). These models are often used to explore the implications of environmental or experimental changes in individual-level demography at the population level, for example how do size-specific harvesting rates influence population dynamics and trait evolution (Wallace, Leslie & Coulson 2013). The most commonly used data-driven models are matrix projection models (MPM), which project discrete population structure (e.g. age or size class) in discrete time. These models are well understood mathematically and there is a well-developed toolbox of techniques for their analysis (Caswell 2001). A major constraint on these models for populations where individuals are characterized by continuous variation is the assumption that individuals can be classified by a small number of discrete states, say for a size-structured population, small, medium and large individuals.

In some animal populations, an individual's fate is often well predicted by their age or life stage (e.g. mature vs. immature), and matrix projection models yield a good description of population-level processes. However, in many populations, a continuous trait such as body mass is a key determinant of performance: all else being equal, larger individuals tend to exhibit greater survival and fecundity, so using a continuous state variable will be more appropriate and often improve the performance of the model (Ramula, Rees & Buckley 2009). In other cases, the whole purpose of developing a model is to understand how a continuous trait impacts ecological and evolutionary processes (Coulson, Tuljapurkar & Childs 2010; Coulson 2012). For example, (Ozgul *et al*. 2010) explored how changes in body mass associated with environmental change mediated an historic shift in the population dynamics of a yellow-bellied marmot (*Marmota flaviventris*) population. In such settings, a framework for working with continuous trait variation is needed.

Easterling, Ellner & Dixon (2000) originally proposed the integral projection model (IPM) as an alternative to matrix projection models for populations in which demographic rates are primarily influenced by a continuously varying measure of individual size or state. Their model was deterministic and density-independent, analogous to a matrix projection model with a constant matrix (Caswell 2001). Since then, IPMs have developed considerably and now there are a wide range of methods available for analysing populations with complex life cycles, time lags, density dependence, environmental stochasticity and where individuals are distributed in space (Childs, *et al*. 2003, 2004; Ellner, & Rees 2006, 2007; Kuss *et al*. 2008; Rees & Ellner 2009; Coulson, Tuljapurkar & Childs 2010; Jongejans *et al*. 2011; Ellner & Schreiber 2012). An R package for parameterizing and analysing some types of IPM is also now available (Metcalf *et al*. 2013).

These mathematically sophisticated papers assume that an IPM has been constructed and parameterized, and provide methods for analysing the resulting model. However, in the development of IPMs, relatively little has been written about: (i) the basic construction of an IPM, and in particular about how different life cycles and census times determine the structure of an IPM and (ii) appropriate statistical analyses for model parametrization, in particular model diagnostics and alternatives to simple parametric models. In this paper, we provide a detailed description of the entire process from data to implementation of the IPM. Specifically we: (i) briefly describe the mathematical background to IPMs for those unfamiliar with the approach; (ii) provide a careful description of how to map your study system onto an IPM, dealing with how the life cycle and census times determine the structure of the IPM and the statistical analysis; and (iii) look at model diagnostics, for the IPM structure, fitted functions and implementation. Finally, we present methods for relaxing some of the common statistical assumptions used for constructing IPMs; we look at more general regression models, and the use of GAM and kernel estimators which allow very flexible descriptions of the mean and distribution of residuals about the fitted demographic models, respectively.

To illustrate the process of building an IPM, we generate artificial data using an individual-based model of the Soay sheep (*Ovis aries*) population on St. Kilda (Clutton-Brock & Pemberton 2004). Throughput the paper, we provide R code for each step, so readers can easily implement an IPM for their study system.

We assume ‘ideal’ data: individuals are reliably marked, accurately measured and can be refound at each census if still alive, and otherwise presumed dead. Many plant studies achieve this ideal, and some animal studies (such as the Soays) come close. Reviewing the specialized tools for imperfect recapture, open populations and similar issues (e.g. www.phidot.org/software/mark) would greatly lengthen the paper, and capture–recapture analysis with continuous covariates is still under development (Langrock & King 2013), so it would be premature to offer advice. We also limit ourselves to model construction and implementation, and say nothing about estimating the uncertainty in estimates or projections. About that, however, we can offer advice: bootstrap. The methods for data resampling, standard errors, etc. in matrix population models (e.g. Kalisz & McPeek 1992; McPeek & Kalisz 1993; Caswell 2001, Ch. 12) are all directly applicable to IPMs.

## Key assumptions and model structure

Individuals within a population typically vary in many different ways, but for simplicity, we assume that variation among individuals is completely summarized by a single quantity *z*, which describes an individual's state (e.g. size, fat stores), and this determines its fate. We often refer to *z* as ‘size’, meaning some continuous measure of body size such as total mass or the log of snout-to-vent length. However, *z* can be unrelated to size – for example, it could be the individual's spatial location in a linear habitat such as a riverbank, or a bird's first egg-laying date. However, *z* must have finite limits: a minimum possible value *L* and a maximum value *U*. Ideally, *z* will be the character or set of characters most strongly linked to individual survival, growth and reproduction, though *z* cannot be, for example, an individual's realized growth rate or fecundity. The premise of an IPM is that individuals with the same current state *z* have the same odds of different future fates and states, but what actually happens involves an element of chance.

The state of the population at time *t* is described by the size distribution *n*(*z*, *t*). Technically, for each time *t*, *n*(*z*, *t*) is a smooth function of *z* such that

The number of individuals with trait value *z* in the interval 
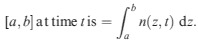
eqn 1

A more intuitive description is that the number of individuals in the size interval [*z*, *z*+*h*] is approximately *n*(*z*, *t*)*h*, and as *h*→0 this approximation becomes exact (the relative error goes to 0). It can be tempting to think of *n*(*z*, *t*) as the number of size-*z* individuals at time *t*, but this is incorrect and can lead to confusion. It is more like the relative frequency of size-*z* individuals, analogous to the bell-shaped curve that characterizes the relative frequency of different possible values in a Gaussian distribution, and its integral from *L* to *U* is the total number of individuals.

Between times *t* and *t*+1, individuals can grow, shrink or die, and they can produce offspring that vary in size. To describe the net result of these processes, we define two functions *P*(*z*′,*z*), representing survival and growth or shrinkage, and *F*(*z*′,*z*) representing the production of new recruits. In both of these, *z* is size at time *t* and *z*′ is size at time *t*+1. For an individual of size *z* at time *t*, *P*(*z*′,*z*)*h* is the probability that the individual is alive at time *t*+1, and its size is in the interval [*z*′,*z*′+*h*] (as with *n*(*z*,*t*) this is an approximation that is valid for small *h*, and the exact probability is given by an integral like eqn 1). Similarly, *F*(*z*′,*z*)*h* is the number of new offspring in the interval [*z*′,*z*′+*h*] present at time *t*+1, per size-*z* individual at time *t*.

It is often convenient to break *P* up into two processes: size-dependent survival and size transitions from *z*→*z*′, for example 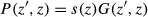
eqn 2where *s*(*z*) is the survival probability, and *G*(*z*′,*z*) describes size transitions. Note that *G*(*z*′,*z*) is not the probability density for the joint distribution of initial and subsequent sizes; rather, it is a family of univariate probability densities for subsequent size *z*′ that depends on initial size *z*. If you are not familiar with probability densities and how they differ from probabilities, see Appendix 1. Examples of *F*(*z*′,*z*) will be presented in following sections.

The net result of survival and reproduction is summarized by the function 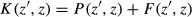
eqn 3called the *kernel* – we will refer to *P*(*z*′,*z*) and *F*(*z*′,*z*) as the survival and reproduction components of the kernel, or as the survival and reproduction kernels. The population at time *t*+1 is just the sum of the contributions from each individual alive at time *t*, 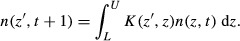
eqn 4

The kernel *K* plays the role of the projection matrix in a matrix projection model (MPM), and eqn 4 is the analogue for the matrix multiplication that projects the population forward in time.

*P* and *F* have to be smooth functions for these definitions to make sense. We have previously assumed that they are continuous (Ellner & Rees 2006), but it is actually sufficient for *P* and *F* to be piecewise continuous. This means that an IPM can include piecewise regression models, such as fecundity that jumps from zero to a positive value once individuals reach a critical ‘size at maturity’.

## From life cycle to model: specifying a simple IPM

The kernels *P* and *F* describe all the possible state transitions in the population, and all possible births of new recruits. But where do these kernels come from? In this section, we answer that question by showing how to translate population census data into a simple deterministic IPM for a size-structured population. We will describe how information on growth, survival and reproduction is combined to make a kernel. As IPMs are data-driven, our aim is to show how to arrive at a model which is both *consistent* and *feasible*. By *consistent*, we mean a model that accurately reflects the life cycle and census regime. By *feasible*, we mean a model that can also be parameterized from the available data. In the case study below, we will then take the next step of fitting specific models to data.

The key idea is that the kernel is built up from functions that describe a step in the life cycle of the species, based on the data about each step. Throughout, we assume that the data were obtained by marking individuals, and following them over their life cycle with evenly spaced censuses at times *t* = 0,1,2,….

**Figure 1:**
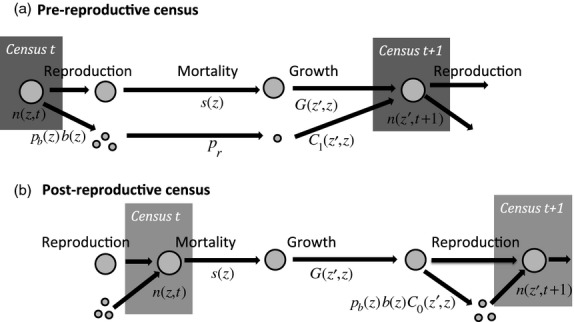
Life cycle diagram and census points for (a) pre-reproductive and (b) post-reproductive census. The sequence of events in the life cycle is the same in both cases. However, the diagrams are different because reproduction splits the population into two groups [those who were present at the last census (large circles), and new recruits who were not present at the last census (small circles)], while at a census time the two groups merge. Reproduction is described by a two-stage process with *p*_*b*_(*z*) being the probability of reproduction and *b*(*z*) being the size-specific fecundity. Each new recruit has a probability *p*_*r*_ of successful recruitment, and its size at the next census is given by *C*_1_(*z*′,*z*). The pre-reproductive census leads to IPM kernel *K*(*z*′,*z*) = *s*(*z*)*G*(*z*′,*z*)+*p*_*b*_(*z*)*b*(*z*)*p*_*r*_*C*_1_(*z*′,*z*) where *C*_1_(*z*′,*z*) is the size distribution of new recruits at age 1 (when they are first censused). The post-reproductive census leads to IPM kernel *K*(*z*′,*z*) = *s*(*z*)*G*(*z*′,*z*)+*s*(*z*)*p*_*b*_(*z*)*b*(*z*)*C*_0_(*z*′,*z*) where *C*_0_(*z*′,*z*) is the size distribution of new recruits at age 0 (when they are first censused). The term *p*_*r*_ is absent in the post-reproductive census because new recruits are censused ‘immediately’ after birth, before any mortality occurs.

We strongly recommend that you begin by drawing a life cycle diagram indicating when demographic processes and census points occur, see Fig. 1. For this first example (Fig. 1a), we assume that at each time step, there is a single census point immediately prior to the next occurrence of reproduction (i.e. there is a *pre-reproductive* census). At that time, you record the size of each individual. At time *t*+1, the population will include survivors from time *t* and new recruits. We assume, for now, that you can assign offspring to parents and therefore can record how many offspring each individual has giving you a data table like this:

**Table:** 

Size *t*	Offspring
3	0
7	5
8	4

Combining this with the size measurements taken at time *t*+1, we end up with a table suitable for statistical analysis:

**Table:** 

Size *t*	Offspring	Survive	Reproduced	Size *t*+1
3	0	0	0	NA
7	5	1	1	8
8	4	1	1	10

where we have added two indicator variables for survival (did the individual survive to *t*+1?) and reproduction (did the individual have any new offspring?).

To define the structure of the IPM, we can begin by ignoring individual size and constructing a model for the dynamics of *N*(*t*), the total number of individuals at census *t* that reflects the life cycle and data. Each individual at time *t* can contribute to *N*(*t*+1) in two ways: survival and reproduction.

1. *Survival*: Having observed how many individuals survive from each census to the next, you can estimate an annual survival probability *s*. At time *t*+1, the population then includes *sN*(*t*) survivors from time *t*.
2. *Reproduction*: We could similarly define a per-capita fecundity *b*, and let *p*_*r*_*bN*(*t*) be the number successful recruits at time *t*+1, with *p*_*r*_ being the probability of successful recruitment. But the data distinguish between ‘breeders’ and ‘non-breeders’, so we can be more mechanistic. Let *p*_*b*_ denote the probability that an individual reproduces, and *b* the mean clutch size among individuals that reproduce. Then, the number of new recruits at time *t*+1 is *p*_*b*_*p*_*r*_*bN*(*t*).

Combining survivors and recruits, we have the unstructured model 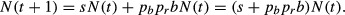
eqn 5The ‘kernel’ for this model is *K* = *s*+*p*_*b*_*p*_*r*_*b*, which is just a single number because at this point, the model ignores the size structure of the population.

The next step is to incorporate how the size at time *t* affects these rates: the probability of surviving, the probability of reproducing and the number of offspring are all potentially functions of the individual's current size *z*: 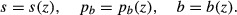


Our prediction of *N*(*t*) can now take account of the current size distribution, *n*(*z*,*t*), 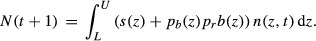
eqn 6At this next level of detail, the kernel is a function of current size *z*, *K*(*z*) = *s*(*z*)+*p*_*b*_(*z*)*p*_*r*_*b*(*z*).

What is missing from model (6) is the size distribution at time *t*+1. To forecast that, we need to specify the size distributions of survivors and recruits. These are given by the growth kernel for survivors, *G*(*z*′,*z*), and the size distribution of recruits, *C*_1_(*z*′,*z*), as described in Section Key assumptions and model structure, giving us the complete kernel 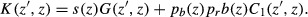
eqn 7for the general IPM (eqn 4). So the structure of the kernel is determined by the life cycle and when the population is censused.

Going back to the life cycle diagram, what would happen to the structure of the kernel if you had conducted a post-reproductive census (Fig. 1b)? The first thing to notice is that order of events has changed. Mortality now occurs before reproduction. This has important implications for the structure of the kernel and for the statistical analysis of the data. For a post-reproductive census, the data file will now look like this:

**Table:** 

Size(*t*)	Offspring	Survive	Reproduced	Size(*t*+1)
3	NA	0	NA	NA
7	5	1	1	8
8	4	1	1	10

The main difference to notice here is that for individuals that die before the next census, there are now NA's in the Offspring and Reproduced column. This has to be so, because the life cycle in this case has mortality occurring *before* reproduction. As a result, the structure of kernel in this case is 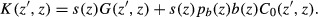
eqn 8

In order to reproduce individuals now have to survive, hence *s*(*z*) is a factor in both the survival and reproduction components of the kernel. The absence of the *p*_*r*_ term is a consequence of censusing the population immediately after reproduction. Newly produced individuals do suffer mortality before their next census at age 1, but this is included in the *s*(*z*) term because already at age 0 they are part of *n*(*z*,*t*) (Fig. 1b). The functions *p*_*b*_(*z*) and *b*(*z*) are now the probability of reproducing, and mean number of offspring produced, for individuals that survive the time step. The NA's in the data table will make sure that you ‘remember’ this, and only data on survivors to fit these functions.

When reproduction occurs just before the next census (as in Fig. 1b), *p*_*b*_ and *b* could be fitted instead as functions of *z*′, the size at the post-breeding census which is also the size when reproduction occurs. This seems more natural, but it leads to a more complicated model. In that approach, the steps to producing size-*z*′ offspring are: survive and grow to some size z^*^, breed or not (depending on z^*^) and if so have *b*(z^*^) offspring, some of which are size *z*′. The fecundity kernel needs to total this up over all possible sizes z^*^, which is: 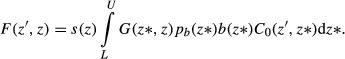
eqn 9Alternatively, the interval between censuses can be broken up into survival/growth (*t* to *t*+τ) and reproduction (*t*+τ to *t*+1) phases with separate kernels: 
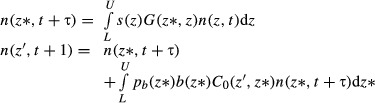
eqn 10

We think that eqn 8 is simpler. When you fit *p*_*b*_ and *b* as functions of *z*, you are in effect letting the data do the integrals with respect to z^*^ for you, because the fitted models will represent the average breeding probability and fecundity with respect to the distribution of possible sizes when reproduction occurs.

The size distribution of new recruits will also vary depending on the timing of the census. In the post-reproductive census, *C*_0_(*z*′,*z*) is the size distribution of recruits at age 0 immediately after their birth (or so we assume). In the pre-reproductive census, recruits were born immediately after the previous census, so they have already undergone a period of growth before they are first observed, and so *C*_1_(*z*′,*z*) is the distribution of new recruits aged 1.

In contrast to the fecundity kernel, the survival kernels in (7) and (8) are the same. Indeed, so long as the demographic models are all based on *z* (initial size), it does not matter where growth falls in the sequence of events, or if growth is instead a continuous process that overlaps with mortality and reproduction. If you know each individual's initial size and whether or not it survived through the next year, those are the data to which *s*(*z*) is fitted. If you know the final size of all survivors, those (initial size, final size) pairs are the data to which *G*(*z*′,*z*) is fitted.

If there are multiple censuses per year, the additional measurements can inform the construction of the kernel, in particular the order of events in the life cycle diagram can reflect the additional information. Multiple censuses also mean that we have to decide when we are going to project the population's state. This will typically be one of the census times but might not, say if we used average or maximum size over several censuses as our measure of size. Alternatively, the annual projection could be broken up in to several census-to-census projections as in eqn 11, like a seasonal matrix projection model. In Appendix 4, we use the Soay sheep case study to present one approach to constructing an IPM when there are several censuses within an annual cycle. In some systems, there may be additional time delays, say because reproduction is better predicted by an individual's state at time *t*−1. This leads to a time-lagged IPM, where the state of the population at time *t*+1 depends the survival and growth of individuals at time *t* and the recruitment of new individuals, which depends on the size structure at time *t*−1 (Kuss *et al*. 2008).

In practice, there are often many possible measures of individual size or state that you could use (the mass or snout–vent length of a crocodile and so on), many possible transformations (log, square root, etc). We cannot emphasize strongly enough the importance of finding the best models to forecast survival, fecundity and growth. This step is like any other statistical analysis of demographic data, so there is no all-purpose recipe, but there are standard tools readily available. For now, we assume that you have made the right choices, but we come back to modelling issues in Section Model diagnostics.

The life cycles considered in this section are just two of the simplest possibilities, and many organisms could not be shoehorned comfortably into either of them. In Appendix 4, we use the Soay sheep system to present a general approach for situations where the sequence of events is more complicated and different data are taken at various times in the year. The important message of this section is that the structure of the IPM is jointly determined by the timing of the census and the order of events in the life cycle, and both these also have important consequences for the statistical analyses to estimate the demographic functions making up the kernel.

## Numerical implementation

The one-variable IPM (4) is easy to implement numerically with a numerical integration method called *midpoint rule* (Ellner & Rees 2006). Define *mesh points z*_*i*_ by dividing the size range [*L*,*U*] into *m* artificial size classes of width *h* = (*U*−*L*)/*m*, and let *z*_*i*_ be the mid-point of the *i*th size class: 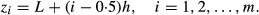
eqn 11The mid-point rule approximation to eqn 5 is then 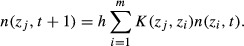
eqn 12

In R (see Box 1), we arrange the *hK*(*z*_*j*_,*z*_*i*_) terms in a matrix called the *iteration matrix*. This allows us to iterate the model by matrix multiplication and use the wide range of numerical tools available for matrices.

**Implementing mid-point rule in R**

A one-variable IPM can always be summarized by the functions *P*(*z*′,*z*) and *F*(*z*′,*z*). So we assume that you have a script that defines functions to calculate their values

P_z1z<- function(z1,z,m.pars) {

# your code, for example

# return(s(z)^*^ G(z1,z))

# using functions s and g that you have defined

}

F_z1z<- function(z1,z,m.pars) {

# your code

}

Here m.pars is a vector of model parameters. The next step is to compute the mesh points

L <- 1; U <- 10 # size range for the model

m <- 100 # m is the size of the iteration matrix

h <- (U-L)/m

meshpts <- L + (1:m)^*^h - h/2

The R function outer makes it easy to compute the iteration matrix

P <- h ^*^ (outer(meshpts, meshpts, P_z1z))

F <- h ^*^ (outer(meshpts, meshpts, F_z1z))

K <- P + F

We put this code inside a function mk_K which has four arguments: the number of mesh points, the parameter vector and the two integration limits, so

mk
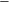
K (nBigMatrix, m.par.true, min.size, max.size)

returns P,F,K calculated using the parameters in m.par.true using nBigMatrix mesh points from size min.size to max.size.

The only drawback to mid-point rule is that it is not very efficient relative to higher-order integration methods, so sometimes it takes a very large iteration matrix to get accurate results. In the section ‘Model diagnostics’ we give some pointers on choosing the size of the iteration matrix and the size limits *L*, *U*, and mention some alternatives to mid-point rule.

## Case study

So far we have looked at translating your study system into an IPM and how to solve the model numerically. In this section, we will put all this together, to show you that building a basic IPM is really pretty straightforward: there is no black magic or black boxes. To do this, we will develop a case study for an idealized animal system, based on published empirical studies. We will simulate data from an individual-based model (IBM) – a simulation that tracks individuals – and analyse the resulting data to build an IPM.

Our example explores the body mass-structured dynamics of an ungulate population: the feral Soay sheep (*O. aries*) of the island of Hirta in the St. Kilda archipelago, off the north-west coast of Scotland. We have chosen to base our example on this population because it has been a major target of research into the dynamics and evolution of wild populations (Clutton-Brock & Pemberton 2004). Although we use simulated data, the IBM was parameterized using the Soay sheep data set. The advantage of using simulated data is that we know the underlying process that generated the data and so can see how various modelling assumptions influence our results. Our aim is to outline a general approach for moving from an individually structured data set to a fully parameterized IPM. The resulting workflow can be applied to almost any life history that can be approximated as a sequence of transitions in discrete time.

## Summary of the demography

We assume that our simulated population is similar to the real Soay sheep population, but with a few important simplifications. The St. Kilda population has been studied in detail since 1985. Each year during this period, newborn individuals are caught, weighed and tagged shortly after birth in the spring, and body mass measurements are taken from approximately half the population each summer during the August catch. Maternity is inferred from detailed field observations and genetic data, while periodic censuses and mortality searches ensure that individual mortality status and population density are very well characterized. Since body mass data on established individuals and new recruits are only available during the August catch, it makes sense to choose this date as our census point to project the dynamics from. Almost all of the mortality in the system occurs during the winter months when forage availability is low and climate conditions are harsh (Clutton-Brock & Pemberton 2004).

These features of the life history and census regime mean that the life cycle diagram for the Soay system corresponds to the post-reproductive census case in Fig. 1b, that is, survival precedes reproduction and individuals are censused prior to the key mortality period. A potential problem with assuming this sequence of events is that only adults that survive from one August catch until the next contribute new recruits to the population, despite the fact that lambing occurs several months earlier in the spring. This is demographically equivalent to assuming that any reproducing individual that dies between giving birth in the spring and the August catch will fail to raise viable offspring. With a handful of exceptions, this is precisely what is observed in the Soay system, so we consider this to be a reasonable assumption.

To keep our example tractable, we will make a number of simplifications: (i) we only consider the dynamics of females; (ii) we ignore the impact of age structure; (iii) we assume that the environment does not vary among years, either as a result of density dependence (e.g. resource limitation) or abiotic factors (e.g. winter weather); and (iv) we assume that Soay females only bear singletons, though in reality they produce twins at a rate 10–15% in any given year. All of these assumptions can be relaxed, although the resulting model is rather more complicated (Childs *et al*. 2011). We work with natural log body mass, based on the analysis of the real data; in Appendix 2, we discuss why log-transformed size is often a good choice for IPMs. The individual-based model is described fully in Appendix 3, and the R code is in Ungulate Simulate IBM.R in the Supporting information.

## Demographic analysis

The results of the IBM simulation are stored in an R data frame (called sim.data), with columns z, z1 containing the sizes in year *t* and *t*+1; indicator variables for survival (Surv = 1 if survived), reproduction (Repr = 1 if reproduced) and recruitment (Recr = 1 if recruited); and a final column for recruit size (Rcsz). We should check the data set carefully to make sure it has the structure we are expecting.

**Table:** 

z	Surv	z1	Repr	Recr	Rcsz
3.07	0	NA	NA	NA	NA
3.17	1	3.24	1	1	2.47
3.02	1	3.17	0	NA	NA
2.92	0	NA	NA	NA	NA
3.14	1	3.25	0	NA	NA
3.06	1	3.00	0	NA	NA

Inspection suggests this data set is as we expect. For example, individuals that die (Surv=0) have missing values (NAs) in all the other columns, and the individuals that survive (Surv=1) but fail to reproduce (Repr=0) have a sequence of missing values for the three remaining variables describing offspring recruitment.

Since we know how the data were generated, we can fit the ’right’ models to the data; we will deal with methods for model criticism later. Survival is modelled by a logistic regression, so we fit it by mod.Surv <- glm(Surv ∼ z  , family = binomial, data = sim.data)

The same is true for whether or not a female reproduced (Repr), and whether or not that lamb survived to recruit into the population their first summer (Recr), mod.Repr <- glm(Repr ∼ z, family = binomial, data = sim.data)

mod.Recr <- glm(Recr ∼ 1, family = binomial, data = sim.data)

Note that mod.Recr does not have any dependence on mother's size *z*, so it is estimating a single number: the recruitment probability. The subsequent sizes of new recruits and of surviving adults are fitted by linear regression, mod.Grow <- lm(z1 ∼ z, data = sim.data)

mod.Rcsz <- lm(Rcsz ∼ z, data = sim.data)

All the models were fitted using the sim.data data frame, which was possible as the NAs ensure that only appropriate individuals are included in each analysis.

**Figure 2:**
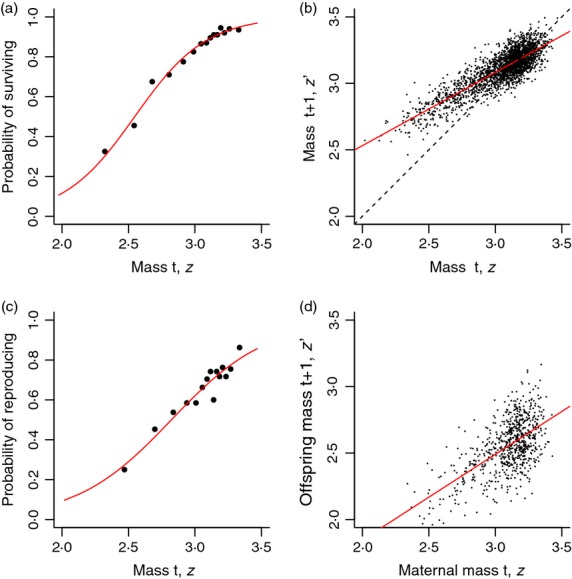
The main mass-dependent demographic processes in the Soay sheep life cycle, as a function of current size, *z* in year *t*. (a) The probability of survival, (b) female mass in the next summer census (August catch), (c) the probability of reproduction and (d) offspring mass. Source file: Ungulate Calculations.R in Supporting Information.

The fitted models are summarized in Fig. 2, and all of the models look reasonable as expected. Having fitted the various models, we then store the parameter estimates in m.par.est, using the same order and names as the parameter vector from the IBM (m.par.true).

m.par.est <- c(

## survival

surv= coef(mod.surv.glm)

## growth

grow= coef(mod.grow)

grow.sd = summary(mod.grow)$sigma

## reproduce or not

repr= coef(mod.repr)

## recruit or not

recr = coef(mod.recr)

## recruit size

rcsz = coef(mod.rcsz)

rcsz.sd = summary(mod.rcsz)$sigma)

names(m.par.est) <- names(m.par.true)

## Implementing the IPM

The next step is to write down the kernel and check whether its formulation matches our knowledge of the life cycle and the data collection protocols, 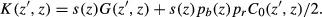
eqn 12

In this instance, both the survival and reproduction kernel components contain the *s*(*z*) term, because the main period of mortality occurs prior to reproduction. The survival component includes the growth kernel *G*(*z*′,*z*); as in the example below, *G* is often specified in terms of the conditional mean, variance and distribution family of subsequent size. The reproduction component is simply the product of the reproduction function, *p*_*b*_(*z*), the probability of survival from spring until summer, *p*_*r*_, and the conditional offspring size function, *C*_0_(*z*′,*z*). The factor of 1/2 appears because we are tracking the dynamics of females and have assumed an equal sex ratio.

We are going to use the approach given in Box 1, so we need to specify the *P*(*z*′,*z*) and *F*(*z*′,*z*) functions

## Define the survival-growth kernel

P
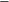
z1z <- function (z1, z, m.par) {

return(s
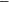
z(z, m.par) ^*^ G_z1z(z1, z, m.par))

}

## Define the reproduction kernel

F
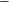
z1z <- function (z1, z, m.par) {

return( s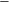 z(z, m.par) ^*^ pb_z(z, m.par) ^*^ (1/2) ^*^ pr_z(m.par) ^*^ C_0z1(z1, z, m.par) )

}

These are just R translations of the two kernel components, and each function is passed a numeric vector, m.par, that holds the parameter values for the underlying demographic regressions. In order to complete the implementation of our model, we next need to define the various functions called within P_z1z and F_z1z. These follow in a very intuitive way from the statistical models we fitted to data. For example, the function describing the probability of survival is

s
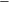
z <- function(z, m.par) {

linear.p <- m.par["“surv.int"] + m.par[“"surv.z"] ^*^ z

# linear predictor

p <- 1/(1+exp(-linear.p))

# inv-logistic trans

return(p)

}

pb
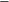
z <- function(z, m.par) {

linear.p <- m.par["“repr.int"] + m.par["“repr.z"] ^*^ z

p <- 1/(1+exp(-linear.p))

return(p)

}

These functions extract the appropriate parameters, calculate the linear predictor at each value of *z* and then transform this onto the probability scale. For s_z and pb_z, the output is a number, the probability of the event in question, but *G*(*z*′,*z*) needs to evaluate a function, the probability density function for size at the next census. To do this, we use the regression of *z*′ on *z* to calculate a sheep's expected size at the next census given its current size. The standard deviation of *z*′ is determined by the scatter about the regression line in Fig. 2b, which we also estimate from the fitted regression. Finally, because the deviations about the fitted line are assumed to follow a Gaussian distribution, we calculate the probability density of observing a sheep of size *z*′ given its current size using dnorm.

G
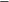
z1z <- function(z1, z, m.par) {

mu <- m.par[“"grow.int"] + m.par["“grow.z"] ^*^ z

# mean size next year (z1)

sig <- m.par["“grow.sd"]

# sd about mean

p.den.grow <- dnorm(z1, mean = mu, sd = sig)

# pdf for size z1

return(p.den.grow)

}

Finally, we calculate the probability density function for recruit size at the next census, given parental size in the current census, which uses the same approach.

C
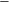
0z1z <- function(z1, z, m.par) {

mu <- m.par["“rcsz.int"] + m.par[“"rcsz.z"] ^*^ z

# mean size of recruits next year

sig <- m.par["“rcsz.sd"]

# sd about mean

p.den.rcsz <- dnorm(z1, mean = mu, sd = sig)

# pdf for offspring size z1

return(p.den.rcsz)

}

The final step in implementation is choosing the size range and number of mesh points. Because large individuals tend to shrink and their offspring are much smaller than themselves (Fig. 2b,d), the upper limit just needs to be slightly larger than the largest observed size, and we set *U* = 3·55. The smallest individuals tend to grow, but they have a small chance of breeding and having offspring who are even smaller than themselves. To make sure that the IPM includes those individuals, we can compute the mean offspring size for the smallest observed size (*z*≈2) and subtract off two standard deviations of offspring size

m.par.est[“"rcsz.int"] + m.par.est["“rcsz.z"]^*^2 − 2^*^ m.par.est["“rcsz.sd"]

1.523043

So we take *L* = 1·5. The total size range is *U*−*L*≈ 2 units on log scale. We will use 100 mesh points so that the increment between mesh points is ≈0·02 units on log scale, which is about a 2% difference in body mass, see Section Model diagnostics.

## Basic analysis

**Figure 3:**
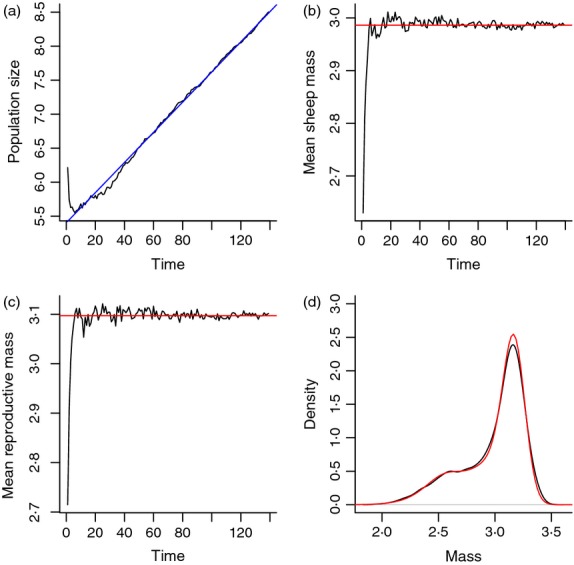
Simulation of the Soay IBM showing (a) log population size, (b) mean sheep size and (c) mean size at reproduction plotted against time. In (d), we have plotted the density estimates for size at the end of the simulation. The red lines are calculated quantities from the estimated IPM, the blue line in (a) is the fitted regression model. Source file: Ungulate Calculations.R in Supporting Information.

There is no density dependence in the Soay IBM, so we expect the population to grow or shrink exponentially and this is indeed what we find, Fig. 3a. The finite growth rate of the population (*λ*) estimated from the simulation by regressing log population size against time is ∼ 1·022, so the population is growing by about 2% each year in the IBM. We can estimate the growth rate using the IPM as follows: (i) use the mk_K function with the estimated model parameters m.par.est to make an iteration matrix; (ii) use the eigen function to compute the dominant eigenvalue of this matrix, which is our estimate of the population growth rate (Easterling, Ellner & Dixon 2000). When we do this, we find that the fitted IPM predicts a *λ* of 1·022, the observed population growth rate in the IBM. Ellner & Rees (2006) review stable population growth theory, which covers the existence of a unique stable population distribution and asymptotic growth rate, which a density-independent population converges to from any initial composition.

The fitted IPM can be used to calculate a wide range of statistical summaries of the population (Rees & Rose 2002). For example, in the IBM, both the mean size and mean reproductive size appear to approach a stable value, Fig. 3b,c. Both these expectations are straightforward to calculate from the stable size distribution, *w*(*z*), which can be calculated using the eigen function. This is just a matter of extracting the dominant eigenvector w of the iteration matrix and normalizing it to a discrete probability density function, stable.z.dist.est <- w/sum(w). The vector stable.z.dist.est can be thought of as the frequency distribution for a set of narrow size classes centred at the values in the meshpts vector defined in Box 1. The mean of the stable size distribution is then mean.z.est <- sum(stable.z.dist.est^*^meshpts).

To calculate the stable size distribution of reproductives, we compute w^*^s_z(meshpts)^*^p_bz(meshpts), which is the discrete approximation *w*(*z*)*s*(*z*)*p*_*b*_(*z*), and then normalize this so that it sums to one and can be used as a probability distribution. Note the details of this calculation depend on the life cycle and census times. In both cases, the IPM calculation provides an excellent description of the individual-based simulation, red lines in Fig. 3b,c.

What if we want to calculate other moments of the stable size distribution, such as the variance, 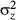
? The same logic applies, and with *w*(*z*) and the mean size, 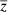, already calculated this is straightforward. For example, the variance can be written as 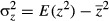
eqn 14where *E*(*z*^2^) is the expected value of *z*^2^ with respect to the normalized stable size distribution. Using the vectors defined above, the R code for this calculation is var.z.est <- sum(stable.z.dist.est ^*^ meshpts 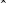 2) − mean.z.est 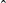 2

var.z.est

[1] 0.07855819

That is, we calculate *E*(*z*^2^) by multiplying each squared mesh point 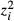 by the proportion of individuals in the size range [*z*_*i*_−*h*/2, *z*_*i*_+*h*/2] and summing these. We then subtract the square of the mean, 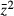, to arrive at the variance. Not surprisingly, this is very close to the size variance estimated directly from the IBM data (=0·078).

More generally, the expected value of any smooth function of size with respect to the stable size distribution can be approximated in this way: first, evaluate the function at the mesh points, multiply each of these by the corresponding value of the normalized stable size distribution and sum. For example, if you want to know the mean size of female sheep on the untransformed size scale – remember, the Soay model works with log body mass – you just need to apply the exponential function to the mesh points first

mean.z.ari.est <- sum(stable.z.dist.est^*^exp(meshpts))

mean.z.ari.est

[1] 20.57083

This again is very close to the value calculated from the IBM, 20·55. Give the close agreement between the calculations from the IPM and IBM, it is not surprising that the predicted stable size distribution from the IPM closely matches that seen in the simulation, Fig. 3d.

## Model diagnostics

Each population is a unique situation, so developing a good model is an iterative process of probing a candidate model for faults and then trying to resolve them. It is important to double-check a model at all steps, from diagramming the life cycle through its computer implementation.

## Model structure

The Soay kernel 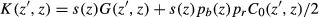
eqn 15is a sentence that you can ‘read out loud’ to see whether it matches what you believe about the population.

The term *s*(*z*)*G*(*z*′,*z*) says:

An individual of size *z* at time *t* can be size *z*′ at time *t*+1 if it survives, and grows (or shrinks) to size *z*′.
  The next term *s*(*z*)*p*_*b*_(*z*)*p*_*r*_*C*_0_(*z*′,*z*)/2 says
Production of new offspring is a multistep process. Starting from the current census, an individual has to survive with probability *s*(*z*) in order to reproduce. Those that survive have a size-dependent probability of breeding, *p*_*b*_(*z*). If it breeds, then it produces a single female offspring with probability 1/2, and the offspring has a probability of surviving to the next census of *p*_*r*_. The size distribution of new recruits that survive, *C*_0_(*z*′,*z*), is dependent on parent size at time *t*.

When you read your kernel aloud in this way, it should match your understanding of the species’ life cycle.

## Demographic rate models

**Figure 4:**
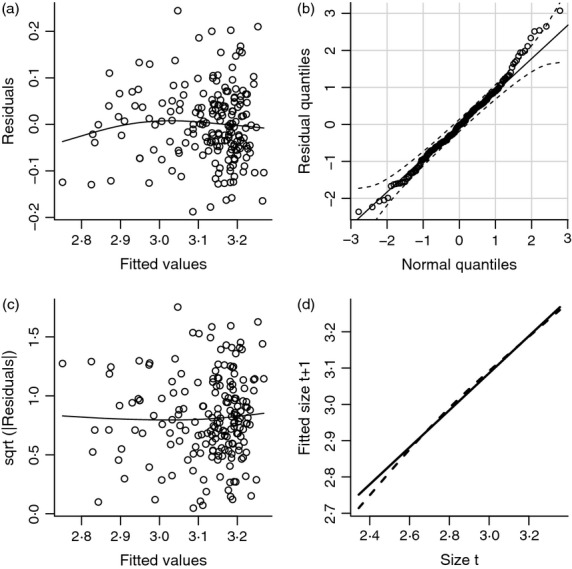
Diagnostic plots for the fitted Soay growth function. (a) Residuals vs. fitted values from the growth model. (b) Residual normal Q-Q plot. (c) Scale-location plot. (d) Expected size at t+1 under linear model (continuous line) and spline model (dashed line). See text for details. Source file: Ungulate Calculations.R in Supporting Information.

The functions that make up the kernel are statistical models whose assumptions about the data can be assessed using standard model diagnostics. Statistical modelling choices are often based on tradition, such as logistic regression for survival. But tradition is often a reflection of what was computationally feasible many decades ago. Modern computers (and the advent of R) give us much more flexibility to let the data dictate the form of demographic models and to carefully test the adequacy of simple models. Figure 4 shows a few simple diagnostics for the Soay growth model *G*(*z*′,*z*), again using the IBM artificial data in the data frame sim.data. The growth model is a linear regression, mod.grow < lm(z1 ∼  z, data = sim.data), here using a subset of the data so that growth is estimated from observations on about 190 individuals. We also calculated the fitted values, residuals and standardized residuals, which will be used later

zhat <- fitted(mod.grow)

resid <- residuals(mod.grow)

sresid <- rstandard(mod.grow)

The first three panels in Fig. 4 are similar to what you would get from R's built-in diagnostics for a linear regression using plot(mod.grow). But we prefer to do it ourselves, so that we can use features from other packages (mgcv, car).

1. Residuals should have constant variance and no trend in mean; plotting residuals vs. fitted values (panel a) provides a visual check on these properties. The plotted curve is a fitted nonparametric spline curve, using the gam function in the mgcv package (Wood 2011). It hints at the possibility of a small nonlinear trend, but this may be driven by a few points at the left (and since the data come from the IBM, we know that the underlying growth model really is linear).
2. Residuals should be Gaussian. A quantile–quantile plot (panel b), using the qqPlot function in the car library, supports this. Perfectly Gaussian residuals would fall on the 1:1 line (solid). The dashed lines are a 95% pointwise confidence envelope, so we should worry if more than 5% of points fall outside the envelope or if any points lie far outside it. In this case, the assumption that the residuals follow a Gaussian distribution seems reasonable. As a further check, we can test for statistically significant departures from Gaussian distribution
  shapiro.test(resid)
  Shapiro–Wilk normality test
  data: resid
  W = 0.9904, p-value = 0.2438
  This confirms what we know: the distribution of residuals about the fitted growth curve is Gaussian.
3. A better check for constant error variance is a scale-location plot (panel c). The plotted points are the square root magnitude of the standardized residuals, and the curve is again a fitted spline. The spline hints at a possible weak trend, so we test for correlation (using Kendall's tau) but find none: cor.test(zhat, sqrt(abs(sresid)), method=“"k") gives *P*=0·40.
4. To follow-up on the hint of nonlinearity in panel (a), we can compare the linear model with a spline fit to the same data (panel d, solid and dashed lines).

The spline in panel (d) suggests that growth is a weakly nonlinear function of size. We know this is not true in this case – it is an accident of random sampling from a linear relationship – but with real data, we would have to decide between the linear and nonlinear models. The statistical evidence is equivocal: a significance test using anova(mod.grow,gam.grow) is marginally non-significant (*P* = 0·089), while AIC slightly favours the nonlinear model (AIC = −411·6) over the linear model (AIC = −410·7).

The AIC difference is small enough that most users would probably select the simpler model. However, its lower AIC means that the nonlinear model is expected to make more accurate predictions (recall that in the frequentist framework AIC is a large-sample approximation to the out-of-sample prediction error, which is why frequentists and agnostics guiltlessly use both AIC and significance tests). Moreover, Dahlgren, Garcia & Ehrlen (2011) have shown that even weak nonlinearities can sometimes have substantial effects on model predictions, so the nonlinear model should be taken seriously. When the statistical evidence for one model over another is equivocal, unless one of the models is strongly favoured based on some underlying biological hypothesis, we believe that the best approach is to try both models and attach most confidence to conclusions that the models agree on. Model averaging is another possibility, but current model averaging approaches are not always effective (Richards, Whittingham & Stephens 2011) and we think that it is often more informative to show the degree of uncertainty by presenting the different results for the range of plausible models.

In an IPM growth model, the residuals are just as important as the mean. In a typical regression analysis, the goal is to estimate the trend represented by the regression line, and inference based on the model will be robust providing the residuals are ‘close enough’ to Gaussian, and the variance is ‘close enough’ to constant. But in an IPM, the scatter around the regression line for growth is also an important part of the model. A smaller growth variance in large individuals might be important for predicting longevity because it keeps big individuals from shrinking, even if it is inconsequential for estimating the effect of size on mean growth rate.

**Figure 5:**
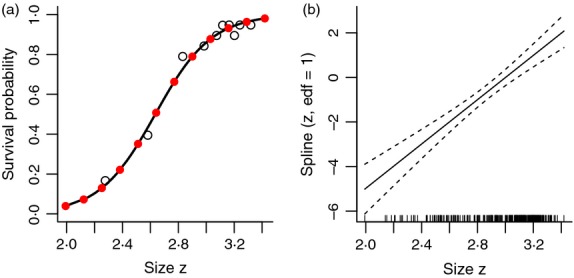
Diagnostic plots for the fitted Soay survival function. (a) Survival as a function of size. The solid curve is the prediction of the fitted GLM (logistic regression); the red circles are the predictions of the fitted nonlinear GAM (nonparametric logistic regression); the open circles are observed survival fraction as a function of mean size for a series of size classes defined by percentiles of the size distribution. (b) The result of plotting the fitted GAM using plot(gam.surv), which shows the fitted spline regression (solid curve) on the scale of the regression model's ‘linear predictor’. If this curve is a straight line (with 1 degree of freedom specifying the slope), the GAM is equivalent to the GLM. In this case, the fitted GAM has 1·0 ‘effective degrees of freedom’, and the linear model is well within the confidence bands on the GAM estimate. Source file: Ungulate Calculations.R in Supporting Information.

Residual plots are less informative for the survival or probability of reproduction models, because all observed values are either 0 or 1, and there is no expectation of Gaussian residuals. One visual check is to compare model predictions with survival rates within size classes (Fig. 5a). And we can again compare the linear model with a nonlinear model using gam (Fig. 5b). In this case, the linear model is supported: it has lower AIC, and the difference between the linear and nonlinear models is minuscule. Further checks are to test for overdispersion and to test whether the fit is significantly improved by adding predictors other than size (e.g. age).

## Implementation: size range and mesh points

It seems natural that a model's size range should correspond to the range of observed sizes, perhaps extended a bit at both ends. Many published studies have used this approach. In our Soay example, we presented a ‘data’ analysis based on a sample of 3000 observations. The minimum and maximum observed sizes in this sample were 1·8 and 3·5, respectively. Because size is on a natural log scale, setting [*L*,*U*] = [1·6,3·7] allows the IPM to include individuals ∼ 20% smaller or larger than any in the sample. However, it is important to check whether a size range chosen in this sort of way is really big enough that individuals are not getting ‘evicted’ from the model. *Eviction* refers to situations where the distribution of subsequent size *z*’ extends beyond [*L*,*U*], so that individuals in the tails of the growth distribution are not accounted for when the model is iterated (Williams, Miller & Ellner 2012).

An effective way to evaluate whether significant eviction is occurring via recruitment or survival-growth is to calculate the probability of eviction as a function of initial size. For example, in the Soay model, the probability that an established size *z* individual gets evicted is the integral of *s*(*z*)*G*(*z*′,*z*) from *z*′ = *U* to *z*′ = ∞. Ideally, this number should be at most a fraction of a per cent for all *z*. What should you do if it is not? Or if you are worried that eviction might still affect evolutionary analyses, because it causes reproductive value to be underestimated? Unfortunately, there is no universally appropriate cure for eviction. A good starting point is to re-examine your growth model, as noted above. If eviction is happening because your growth model has an upper tail that extends beyond what is actually possible, a distribution with tighter limits on growth might fit the data better (e.g. a beta or truncated Gaussian distribution). If your growth model does not have an upper tail beyond *z*′ = *U* but eviction is still occurring, there are several solutions you can try. These are discussed by Williams, Miller & Ellner (2012) so we will not review them here. As always, it is important to consider alternative assumptions and how those affect your conclusions.

Choosing the number of mesh points is a matter of balancing accuracy against computation speed. The number of mesh points *m* required for accurate results depends on the kernel. An integral computed by mid-point rule will be accurate if the function being integrated is close to linear on intervals of length *h* = (*U*−*L*)/*m*. For IPMs, this means that *h* needs to be small compared to the standard deviation of offspring size and to the standard deviation of growth increments *z*′−*z*. If neither of these is too small, then a simple but effective approach is to start with a reasonable value of *m*, say 50, and increase it until numerical results stabilize to the accuracy you need. But if either the standard deviation of offspring size or the growth increments *z*′−*z* is small, direct application of mid-point rule might lead to a very large matrix and to calculations that run slowly or crash when memory runs out.

Small variance in growth increments is particularly problematic. In practice, it arises for long-lived, slow-growing species. One option then is to use computational methods that can deal with large matrices. For example, the dominant eigenvalue and corresponding eigenvectors can be found without also calculating all the others as eigen does. Ellner & Rees (2006) explain how to do this by iteration, and Dawsons (2013) explains how to do this by calling ARPACK routines from within R. A second option is to use more accurate numerical integration methods (Dawson 2013). The third, and likely best, option is to use methods for sparse matrices. Linear algebra functions for sparse matrices (such as those in R's spam and Matrix libraries) only store and work with the nonzero entries in a matrix. When growth is slow and nearly deterministic, the IPM kernel will be structured like a Leslie matrix, with zero or tiny values except in a small strip near the top (fecundity) and just below the diagonal (survival). Zeroing out transitions that do not really occur (e.g. if h^*^K[j,i]<10^−9^) will result in a very sparse matrix, and using sparse matrix methods will speed up the computations enormously. Sparse matrix methods can also be useful in more complex models where individuals are cross-classified by multiple traits (Ellner & Rees 2006).

## Fitting more flexible demographic models

In the Soay example, we have assumed that all demographic processes can be described by simple parametric models, indeed all the models fitted were linear or generalized linear models with constant variance. Many empirical applications of IPMs have found these models to be adequate, but some cases need more flexibility. In this section, we briefly tour some useful approaches, going from simple (variable transformation) to complex (nonparametric estimates of growth variability) but without the need for complex coding by the user. To be concrete, we will think about parameterizing a growth kernel *G*(*z*′,*z*) depending only on individual size, by specifying the conditional mean *m*(*z*) = *E*[*z*′|*z*] and the pattern of individual variation around the mean. In a sense, this is just regression analysis, but as we noted above, it is also important to carefully model the between-individual variation in growth.

## Transforming variables

One long-standing approach is to seek a variable transformation such that the transformed data are fitted by a simple linear model. Log transformation is one example, and it has been sufficient in many IPMs to date. When that fails, a power transformation is sometimes effective, *z* = *x*^*λ*^ where *x* is the raw size measurement (e.g. Bruno *et al*. 2011). Maximum likelihood can be used to find a good value of *λ*. For the linear model *y*^*λ*^∼*a* + *bx*^*λ*^ + Normal(0,σ^2^) with *x*,*y*,*λ*>0, the profile negative log-likelihood of *λ* is (Box & Cox 1964, p. 215) bcNLL=function(lambda,x,y) {

xl <- x
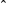
lambda; yl <- y 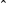 lambda

fit <- lm(yl^∼^xl); s2hat <- mean(fit$residuals 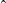 2)

return(0.5^*^length(x)^*^log(s2hat/lambda 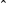 2) − (lambda-1)^*^sum(log(y)))

}

In the following test case, artificial data are created for which 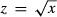 follows a standard linear regression model

z <- runif(N,1,10); z1 <- 1 + 0.5^*^z + 0.2^*^rnorm(N)

x <- z 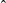 2; x1 <- z1 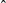 2

To estimate *λ*, we minimize bcNLL with x=size in year *t*, y=size in year *t*+1, lambdaHat <- optimize(bcNLL,c(0.01,3),x=x,y=x1)$minimum

This gives *λ* close to 0·5, as it should (mean = 0·495 and 90% of values between 0·37 and 0·62 with N=100 data points). The code is BoxCoxExample.R. Note, this is *not* equivalent to the boxcox function in R's MASS library, which transforms only the dependent variable. In an IPM growth kernel, the independent variable is also size.

If a transformed variable is used in the growth kernel, it is simplest to use that transformation for the entire model. However, other demographic models can still be fitted on other scales. For example, let *x* be measured size, and suppose that a growth kernel is fitted for *z* =  log *x* but survival is fitted well by logistic regression on untransformed size, *logit*(*s*(*x*)) = *a*+*bx*. The survival function on log scale is then *logit*(*s*(*z*)) = *a*+*be*^*z*^. Transforming growth and offspring size models to different scales is also possible but a bit more complicated, see Appendix 1.

## Non-constant variance

In many cases, a linear model has been adequate for the conditional mean (expected size next year), but the variance in growth is not constant. This variability needs to be modelled so that the IPM produces realistic individual growth trajectories and size distributions.

Linear regression with several different size-dependent variance functions can be done in R using gls (e.g. Ellner & Rees 2006). However, any variance function can be fitted by maximum likelihood. For example, if the mean and standard deviation of *z*′ are both linear functions of *z*, the negative log-likelihood is -sum(dnorm(z1,mean=a+b^*^z,sd=c+d^*^z,log=TRUE)) and the parameter values can be estimated by mle in stats4. Even if your preferred model is available in gls or elsewhere, maximum likelihood has advantages. You can assume a non-Gaussian distribution for growth by substituting a different probability density for dnorm, such as a *t* distribution to accommodate fatter tails. And it is then easy to compare models of varying complexity using AIC, BIC or likelihood ratio tests (e.g. constant vs. linear vs. quadratic dependence on individual size).

## Nonlinear growth: modelling the mean

If your data cannot be transformed so that mean size at the next census is a linear function of present size, a nonlinear mean function (such as a polynomial) can also be fitted by maximum likelihood. But unless you have some biological basis for specifying a particular nonlinear mean function, it is probably preferable to instead fit a flexible nonlinear model whose shape is dictated by the data. This is a strength of R, and many options are available. If the variance appears to be constant, the gam function in mgcv can fit a spline whose degree of nonlinearity is automatically chosen based on the data. For the Soay data, all this takes are require(mgcv); gamGrow <- gam(z1 ∼ s(z),data=sim.data)

**Figure 6:**
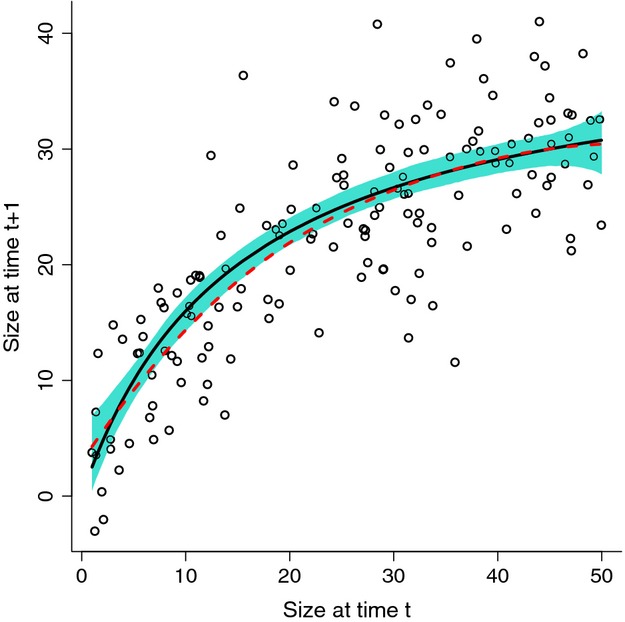
Example of fitting a nonlinear mean growth function with gam. The solid curve (black) is the true mean function 40*z*/(15+*z*), and the circles are one typical simulated data set (Gaussian with standard deviation σ = 5, 150 data points). The dashed curve (red) is the estimate of the mean function from the one simulated data set plotted here. The shaded region (blue) shows the pointwise 5th to 95th percentiles of the fitted mean function over 1000 replicate simulated data sets. Source file: gamExample.R in Supporting Information.

Note that s(z) in the line above is how the mean is specified to be a spline function of *z*, not the survival function. Figure 6 illustrates that this works surprisingly well with a moderate amount of data despite high variance about the mean.

The residuals from the fitted mean curve provide an estimate of the growth distribution's standard deviation, sse <- sum(resid(gamGrow)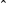 2)

sdhat <- sqrt(sse/df.resid(gamGrow))

In 1000 replicate simulated data sets with the same structure as in Fig. 6 and σ = 5, the mean estimate of σ was 4·98 and 90% of estimates were between 4·5 and 5·5.

There is no formula for the fitted mean function, but it still can be used in an IPM by using the predict method for gam models, as follows.

Gz1
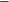
z <- function(z1,z) {

Gdata <- data.frame(z=z)

z1bar <- predict(gamGrow,newdata=Gdata,type=“response")

return(dnorm(z1,mean=z1bar,sd=sdhat))

}

## Nonlinear growth: parametric variance models

Unfortunately, one data set can have several different complications. In the growth model just above, the mean function can be any smooth function, but the residuals are Gaussian with constant variance. It is still essential to check whether growth variability really fits that assumption and to improve the model when it does not.

Size-dependent variance and many non-Gaussian distributions can be fitted using the gamlss package. The mean, variance and up to two additional shape parameters can be fitted as either parametric or nonparametric functions of the independent variables. We cannot review all the options here, but we give one example to illustrate the possibilities. Suppose the growth variability in the last example is instead a *t* distribution with d.f. = 5 and non-constant variance. R's rt function generates a standard *t* distribution with variance 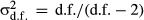, so below we use a *z*-dependent factor scale to create artificial size data with size-dependent variance in growth

z <- runif(250,1,50)

# uniform distribution on [1,50]

z1bar <- 40^*^z/(15+z); scale <- 4^*^exp(-0.5+z/50)

z1 <- z1bar + scale^*^rt(250,df=5)>

**Figure 7:**
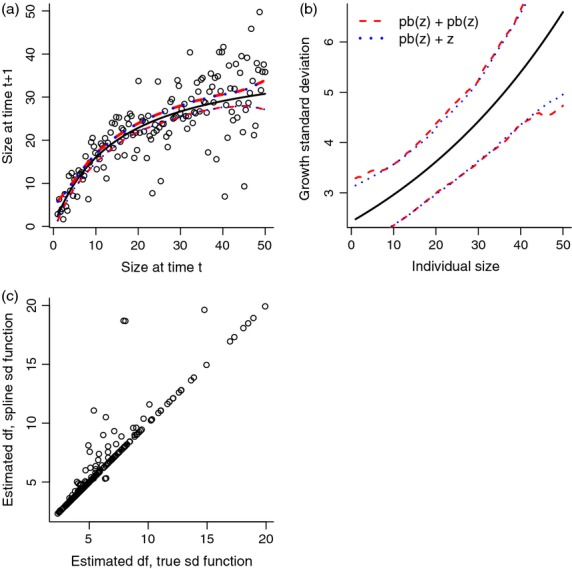
Simultaneously fitting the mean, variance and shape parameter of a *t* distribution. (a) The solid black curve is the true mean function 40*z*/(15+*z*), and the circles are a typical data set. The dashed curves (red) are the pointwise 5th and 95th percentile estimates of the mean function across 250 simulated ‘data’ sets, using nonparametric splines (function pb in gamlss) for the mean and standard deviation functions. The nearly identical dotted curves (blue) are the same, but result from using the correct parametric form of the standard deviation. (b) The solid black curve is the true standard deviation; the dashed and dotted curves are pointwise 5th and 95th percentiles, as in panel (a). (c) Estimates of the shape parameter d.f. in the *t* distribution, with values >20 not shown. Source file: gamlssExample.R in Supporting Information.

Figure 7 shows results from fitting these ‘data’, estimating d.f. (assumed to be size-independent), a nonparametric mean function (cubic splines) and either a nonparametric σ(*z*) (cubic splines) or the true σ(*z*) function (loglinear). The key point is that nonparametric fitting of σ(*z*) was almost as good as (somehow) knowing the true functional form. The only cost is a slightly higher risk of missing the fat tails in the growth distribution (i.e. estimating d.f.≫5, Fig. 7c).

## Nonparametric variance models

As a final level of generality, it is not even necessary to specify a distribution for the growth variability. This may be essential if no standard distribution can capture all the features evident in the growth data, or it may let you avoid a difficult choice between several candidates for the ‘right’ distribution.

Kernel density estimates are convenient for this because they are easy to use in an IPM growth kernel. The fitted density function is just the average of a set of probability densities (typically Gaussian) centred at the residuals. The one subtle point is that replacing each residual by a probability density increases the growth variance, so the residuals should be shrunk to offset this. This sounds complicated, but the code is simple.

For simplicity, we go back to constant variance, using gam to estimate the mean function. The first step is using the residuals to estimate the growth variance

Resids <- resid(gamGrow); # extract residuals

sse <- sum(Resids 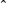 2); # sum of squared errors

sdhat <- sqrt(sse/gamGrow$df.residual)

# estimated Std Dev

The only thing we need from the kernel density estimate is its bandwidth *h*, the standard deviation of the probability densities centred at each residual: h <- bw.SJ(Resids)

The estimated probability density for growth is then the average of Gaussian densities centred at the scaled residuals, dfun in the code below

alpha <- sdhat/sqrt(sdhat
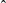
2 + h
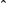
2)

# shrinkage factor

hResids <- alpha^*^Resids

# shrinking the residuals

dfun <- function(z) mean(dnorm(z,hResids,h))

dfun <- Vectorize(dfun)>

Figure 8 shows an example. The ‘data’ follow the model of Fig. 6 except that the growth variance was a *t* distribution with d.f. = 3. Taking the pragmatic approach that the non-Gaussian error distribution will not throw off the estimate of the mean by much, we fitted the mean using gam. The resulting dfun was (correctly) markedly more peaked and fat-tailed than a Gaussian with the same standard deviation. But even with a decent amount of data (here, 150 data points), the estimate for an individual data set can be erratic, especially in the tails. So unless your data set is quite large, a nonparametric estimate of growth variation is probably best used as an exploratory step to identify the qualitative shape of the distribution. The final model can then use a parametric distribution (*t*, beta, lognormal, etc.) that has the right shape.

**Figure 8:** Kernel density estimation, for the same growth function as Fig. 6 but *t*-distributed variation of growth around the mean with d.f.=3. The solid black curve is the true probability density, and for comparison, the dotted curve is a Gaussian with the same variance. The dashed curve (red) is a typical estimate of the density from one set of simulated data on growth of 150 individuals, obtained as described in the text from the residuals of fitting the mean growth function with gam. The shaded area (blue) are the pointwise 5th and 95th percentiles of the density estimates in 500 replicate simulated data sets on growth of 150 individuals. Source file: kernelExample.R in Supporting Information.
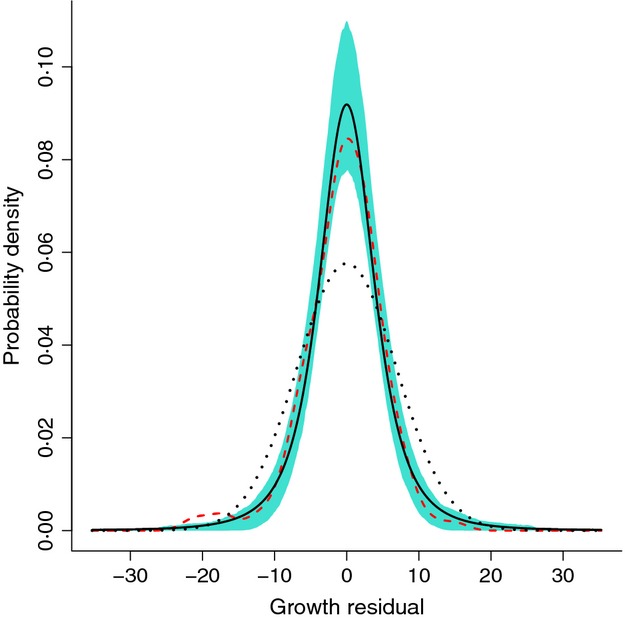

Kernels can still be used if the variance is size dependent. First, estimate the standard deviation function sdG and scale the residuals by their estimated standard deviation, Resids <- resid(gamGrow)/sgG(z). Then compute *h* and hResids as above. The estimate of the growth distribution is then dfun <- function(z) (1/sdG(z))^*^mean(dnorm(z/sdG(z),mean=hResids,sd=h))

Again, this will only be reliable with a very large data set, because the scaled residuals depend on the estimated mean and standard deviation functions.

## Recommendations and extensions

For the beginner just starting out building IPMs, we have two key recommendations:

1. Recommendation 1: Always draw a life cycle diagram like our Fig. 1, which summarizes the biology of the system, when the censuses occur, and has the demographic functions indicated. Failing to do this can result in a model that does not represent your study system making any conclusions drawn difficult to justify. We believe this is likely to be the commonest and most easily made mistake when starting out building IPMs and so recommend all publications using IPMs include a life cycle diagram like our Fig. 1 either in the main text or online.
2. Recommendation 2: Explore your model thoroughly using model diagnostics to test the model structure, whether the demographic models (*s*(*z*) etc) are appropriate, and whether your conclusions are robust to the details of how the model is implemented (e.g. the size range and number of mesh points). We are great believers in ‘suck it and see’, so, for example, if your data does not unequivocally distinguish between two possible growth models then use both and compare the results. If you have used the code suggested in Section Implementing the IPM, exploring alternative growth models only involves changing the definition of G_z1z and rerunning your analysis. This approach to understanding how the various assumptions influence your results will allow you to understand your system better and also determine which conclusions are robust and which need to be interpreted with caution.

The simple IPM we have described is appropriate for some systems (e.g. Wallace, Leslie & Coulson 2013), but it will often be the case that the biology of the system will be more complicated. Since the introduction of IPMs (Easterling, Ellner & Dixon 2000), numerous extensions have been developed (Table1). These extensions can be divided into two categories, those dealing with aspects of the environment in which the population occurs, dealing with say spatial or temporal variation in the quality environment, and those dealing with more complex life histories, for example dormant states or stage structure where individuals are cross-classified by stage (e.g. juveniles and adults) and a continuous state (e.g. size). When building complex IPMs, our advice would be to start simple and add complexity when you are happy with your simpler analysis. For example, when building a stochastic environment IPM for Soay sheep, we would recommend building a constant environment model first, as we have done, and then extending this so the kernel varies from year to year (Childs *et al*. 2004; Rees & Ellner 2009).

**tabel 1:** Applications extending the basic IPM to deal with complications arising from the biological properties of the system

Extension	References
Age structure	(Childs *et al*. 2003), Ellner & Rees (2006)
Discrete states	(Rees *et al*. 2006), Hesse, Rees & Mueller-Schaerer (2008), (Miller *et al*. 2009), Williams (2009), Jacquemyn, Brys & Jongejans (2010)
Stage structure	(Ozgul *et al*. 2010), (Bruno *et al*. 2011), (Childs *et al*. 2011), (Ozgul *et al*. 2012)
Time lags	(Kuss *et al*. 2008)
Multispecies interactions	Rose, Louda & Rees (2005), (Miller *et al*. 2009), Adler, Ellner & Levine (2010), Hegland, Jongejans & Rydgren (2010), (Bruno *et al*. 2011)
Costs of Reproduction	Rees & Rose (2002), (Miller *et al*. 2009)
Density dependence	Ellner & Rees (2006), (Childs *et al*. 2011), Rebarber, Tenhumberg & Townley (2012)
Environmental covariates	(Metcalf *et al*. 2009), (Miller *et al*. 2009), Jacquemyn, Brys & Jongejans (2010), (Coulson *et al*. 2011), Dahlgren & Ehrlén (2011), Dahlgren, Garcia & Ehrlén (2011), (Nicole *et al*. 2011)
Environmental stochasticity	(Childs *et al*. 2004), Ellner & Rees (2007), Rees & Ellner (2009), Adler, Ellner & Levine (2010), (Dalgleish *et al*. 2011)
Demographic stochasticity	Vindenes, Engen & Saether (2011); Vindenes, Saether & Engen (2012)
Space	Adler, Ellner & Levine (2010), (Jongejans *et al*. 2011), Ellner & Schreiber (2012)
Eco-evolutionary dynamics	Coulson, Tuljapurkar & Childs (2010), (Bruno *et al*. 2011), (Coulson *et al*. 2011), Smallegange & Coulson (2013)

IPM, integral projection models.

Having constructed and parameterized your IPM, many different types of analyses are possible. For a discussion of the general mathematical properties of IPMs, see Ellner & Rees (2006, 2007); sensitivity and elasticity analyses are presented in Easterling, Ellner & Dixon (2000), Ellner & Rees (2006) and Rees & Ellner (2009); life table response experiments in Williams & Crone (2006) and Rees & Ellner (2009); evolutionary demography, the calculation of ESSs and selection gradients, in Rees & Rose (2002), (Childs *et al*. 2003, 2004), (Rees *et al*. 2006), (Metcalf *et al*. 2008), (Childs *et al*. 2011); and the calculation of quantitative genetics quantities in Coulson, Tuljapurkar & Childs (2010). But really there is no limit to what you can do. A carefully constructed and critically evaluated IPM is a virtual population that you can manipulate at will (metapopulation structure? restrictions on harvesting? less rain or more floods? why not?), then enumerate and measure completely for as long as you like, without writing a grant proposal or recruiting new students.

## Supporting Information

Data S1 Rcode

Appendix S1 Probability densities vs. probabilities, and moving a kernel from one measurement scale to another.

Appendix S2 Why IPMs often use log-transformed size.

Appendix S3 Description of the Soay individual-based model.

Appendix S4 Constructing IPMs when more than one census is available.
