# Supplementary material for: Building integral projection models: a user's guide
Source: J Anim Ecol. 2014 Jan 20;83(3):528–45. doi: 10.1111/1365-2656.12178 (PMC4258094; doi:10.1111/1365-2656.12178)
Supplement: Appendix S1 — Probability densities vs. probabilities, and moving a kernel from one measurement scale to another. [file jane0083-0528-SD2.pdf]

Appendix S1: Probability densities vs. probabilities, and moving a kernel from one measurement scale to another

M. Rees, D.Z. Childs, and S.P. Ellner.
Building Integral Projection Models: a User's Guide.
Journal of Animal Ecology, 2014

Probability density functions are extensively used in IPMs and so it is important to understand the difference between *probability densities functions* and *probabilities* which are used to define matrix projections models (MPM). For example, in a size-structured matrix model, the projection matrix entry a_{32} representing transitions from size-class 2 to size-class 3, is the product of the survival probability s_2 for size-class 2, with the probability g_{32} that a survivor grows into size-class 3: $a_{32} = s_2 g_{32}$. In the basic size-structured IPM, we have instead

$$P(z', z) = s(z)G(z', z).$$

Here $s(z)$ is the size-dependent survival probability, just like in the MPM. But $G(z', z)$ is the *probability density* of size z' at the next census for survivors having initial size z.

Probability densities are enough like probabilities that you can often ignore the difference. But sometimes you cannot. In past writings we have sometimes glossed over the differences, for example by calling $G(z', z)$ the probability of growing to size z'. But discussions with other IPM builders have convinced us that this shortcut created much more confusion than it avoided, so here we try to be more accurate.

The density of water is the mass per unit volume of water, such as g/cm^3. To get the mass, you multiply density by volume. Similarly, to get the probability for a given range of body sizes, we have to take the probability density and multiply it by the "volume" of the size range. Because size is one-dimensional, the volume of a size range is its length. For a size range of length h we therefore have

$$\text{Probability that subsequent size } z' \text{ is in } [z', z' + h] \approx G(z', z)h. \tag{S1.1}$$

Why do we have $\approx$ rather than $=$ in equation (S1.1)? Why is it just an approximation? The reason is that the probability density for z' is generally not constant, so the density at z' doesn't apply over the whole size range $[z', z' + h]$. To be exact, we have to use the right density for each size, which is accomplished by integration:

$$\text{Probability that subsequent size } z' \text{ is in } [z', z' + h] = \int_{z'}^{z'+h} G(z', z)\, dz. \tag{S1.2}$$

However, you won't go wrong by thinking of equation (S1.1) as being exactly right, so long as you remember that it only holds for small h (narrow size ranges).

From equation (S1.1) we see one way in which probability densities are like probabilities: they give us the *relative* likelihood of different outcomes. In this case,

$$\frac{\text{Probability that subsequent size } z' \text{ is near } z_1}{\text{Probability that subsequent size } z' \text{ is near } z_2} \approx \frac{G(z_1, z)h}{G(z_2, z)h} = \frac{G(z_1, z)}{G(z_2, z)} \tag{S1.3}$$

so long as the same definition of "near" is used at z_1 and z_2. This is the best way to think about the intuitive meaning of probability density: it tells us the *relative* probability of two equal-length (small) ranges of the variable, but not the absolute probability of any particular value or size range.

In the same way, regardless of how often we or anybody else has said otherwise, the fecundity kernel $F(z', z)$ is not the number of size-z' offspring produced by a size-z parent. Rather, as in equation (S1.1), $F(z', z)h$ is (for small h) the number of offspring in the size range $[z', z'+h]$ produced by a size-z parent, and $F(z_2, z)/F(z_1, z)$ is the relative frequency of offspring with sizes near z_2 and z_1.

This difference between probability and probability density is important when you need to move a kernel from one scale of measurement to another. For example, suppose that your data lead you to fit a growth model using a linear ("untransformed") size measure u, but for everything else log-transformed size works better, so you decide to use $z = \log(u)$ as your state variable. Then you need to take the growth model $G(u', u)$ and express it as a growth model on log-scale, $\tilde{G}(z', z)$.

Because G is a probability density for future size (conditional on current size), a change of variables for G has to be made using the change of variables formula for probability densities. The general recipe is as follows. Let f be the function that gives u (the scale on which G was fitted) as a function z (the scale that the IPM uses). In the example above, $z = \log(u)$ so $u = e^z$ and the function f is $f(z) = e^z$. Then the growth kernel on the scale of the IPM is

$$\tilde{G}(z', z) = \left.\frac{df(z)}{dz}\right|_{z=z'} G(f(z'), f(z)) \tag{S1.4}$$

To implement this in an IPM, we recommend writing a function that computes $G(u', u)$ on the scale where G was fitted, and a second function that computes $\tilde{G}$ using equation (S1.4).

Equation (S1.4) applies also to functions of a single variable. The situation is especially simple for a function of just initial size z: $\tilde{s}(z) = s(f(z))$. For a function of z' such as a parent-independent offspring size distribution,

$$\tilde{C}(z') = \left.\frac{df(z)}{dz}\right|_{z=z'} C(f(z')) \tag{S1.5}$$
