# Supplementary material for: Building integral projection models: a user's guide
Source: J Anim Ecol. 2014 Jan 20;83(3):528–45. doi: 10.1111/1365-2656.12178 (PMC4258094; doi:10.1111/1365-2656.12178)
Supplement: Appendix S2 — Why IPMs often use log-transformed size. [file jane0083-0528-SD3.pdf]

Appendix S2: Why IPMs often use log-transformed size

M. Rees, D.Z. Childs, and S.P. Ellner.
Building Integral Projection Models: a User's Guide.
Journal of Animal Ecology, 2014

It is common practise to adopt a log transformation of size when building a new IPM. Why is this? The short answer is that it very often "works", in that log transformation results in a linear growth model in which the error variance (i.e., the variation in growth) ends up independent of size. This is an assumption of linear regression that ensures the estimated parameters are as precise as they can be. More importantly, it means that to model growth we do not have to resort to more sophisticated methods that require additional parameters to model the size-variance relationship. And in cases where growth variance still depends on size after log transformation, the dependence is often weak, so that a simple linear or exponential model with just one additional parameter is adequate. Another practical advantage of using a log transformation is that it avoids the possibility that the IPM generates individuals with negative sizes: you always have a positive size no matter where you lie on a log transformed scale.

The log transformation also makes biological sense when using a linear model to describe growth. For the moment let u denote some absolute measure of size and $z = \log u$, and assume that growth is completely deterministic. Fitting a linear model using absolute size, $u' = A + Bu$, the growth increment $\Delta u = u' - u$ is a strictly decreasing or increasing function of size. That is, $\Delta u = A + (B-1)u$. This is a decreasing function of size if individuals exhibit determinate growth ($B < 1$). However, in many species we observe a humped relationship between the absolute growth increment and size, so the relationship between size and age is sigmoidal. This is precisely the relationship that emerges if we instead assume that the expected change in log size is a linear function of log size and therefore fit a linear regression to successive values of log size, $z' = a + bz$. For species with determinate growth ($b < 1$), this implies that the relative growth rate $\log(u') - \log(u) = z' - z = a + (b-1)z$ is a decreasing function of size. For this model the relationship between the absolute growth increment and size is $\Delta u = e^a u^b - u$. This is hump-shaped when $b < 1$.

Growth is a complex phenomenon, reflecting patterns of resource availability, competition and life history allocation decisions. However, one fairly general explanation for the hump-shaped pattern arises from a consideration of energy acquisition and maintenance costs. All else being equal, larger individuals typically acquire more resources than smaller conspecifics, which means they have more energy available to spend on growth, reproduction and maintenance. When individuals are small, maintenance costs increase slowly with size relative to acquisition, resulting in a positive relationship between size and absolute growth rate. Later in life when individuals are large, maintenance costs increase more rapidly with size relative to acquisition, leading to a negative relationship between size and growth. Such ideas can be formalized using dynamic energy budget theory.

Ultimately, the choice of growth model should be guided by the data: the final model should produce good model diagnostics while avoiding over-fitting the data. This can be done, when need be, by fitting different demographic models on different scales of measurement, e.g. fitting a growth model to the log of size and survival as a function of size or square-root size. The IPM has to run on just one size scale, so it is important to know how to move a demographic model from one scale to another, as we describe in Appendix S1.
