# Supplementary material for: Building integral projection models: a user's guide
Source: J Anim Ecol. 2014 Jan 20;83(3):528–45. doi: 10.1111/1365-2656.12178 (PMC4258094; doi:10.1111/1365-2656.12178)
Supplement: Appendix S3 — Description of the Soay individual-based model. [file jane0083-0528-SD4.pdf]

Appendix S3: Description of the Soay individual-based model

M. Rees, D.Z. Childs, and S.P. Ellner.
Building Integral Projection Models: a User's Guide.
Journal of Animal Ecology, 2014

The IBM was parameterised from a series of linear and generalised linear models fitted to the field data that describe survival, growth and components of recruitment as functions of individual body mass (DZC, *unpublished analysis*).[1] This ensures that the demography of the simulated population is similar to the real Soay sheep population, yet that we know the true parameters underpinning the data. We adjusted the intercept of survival function so that the population growth rate $\lambda \approx 1.01$.

The IBM is implemented as follows. First, for each individual in the current summer population we simulate a Bernoulli random variable, $Surv \sim Bern\,(s(z))$, where $s(z)$ is the size-dependent probability of survival estimated from a logistic regression, and therefore has the form $s(z) = (1 + e^{-\nu_S})^{-1}$, where $\nu_S = -9.65 + 3.77z$. Following the survival phase we allow the surviving ($Surv = 1$) individuals to grow by simulating a Gaussian random variable, $Z1 \sim Norm(\mu_G(z), \sigma_G)$, where $\mu_G(z) = 1.41 + 0.56z$ is the expected mass of an individual next summer given their current size and σ_G is the standard deviation ($= 0.08$) of the conditional size distribution, estimated from a linear regression.

Following survival and growth of established individuals, we simulate a sequence of three processes to add new recruits to the population. For each surviving individual we simulate a Bernoulli random variable which captures reproduction, $Repr = Bern\,(p_b(z))$, where $p_b(z)$ is the size-dependent probability of reproduction estimated from a logistic regression. This function is of the form $p_n(z) = (1 + e^{-\nu_n})^{-1}$, where $\nu_n = -7.23 + 2.60z$. Since we assume that a single lamb is born at each reproductive event, the next step is to simulate a Bernoulli random variable, $Recr \sim Bern\,(p_r)$, for each of the surviving and reproducing ($Repr = 1$) individuals that describes the recruitment of their offspring to the established population next summer. The probability of recruitment p_r is the constant. However, for consistency we estimated it from a logistic regression so that it has the form $p_r = (1 + e^{-1.93})^{-1}$. Finally, we assign a mass to the recruited individuals ($Recr = 1$) by simulating a Guassian random variable, $Rcsz \sim Norm(\mu_c(z), \sigma_c)$, where $\mu_c(z) = 0.36 + 0.71z$ is the expected mass of a recruit given their mother's mass, estimated from a linear regression of offspring summer mass against maternal mass *in the previous summer*, and σ_c is the standard deviation ($= 0.16$) of the conditional size distribution.

Starting with an initial population density of 500, we simulated the population until the density reached 5000 individuals and then selected a random sample of 3000 individuals from the simulated dataset to be used in the following analysis. The R code for the demographic functions and the IBM simulation can be found in `Ungulate Demog Funs.R` and `Ungulate Simulate IBM.R`, respectively. The code for running everything and carrying out the analysis we discuss next is store in `Ungulate Calculations.R`.

[1]The data analysis included year-to-year parameter variation; the parameter values reported here describe an average year.
