# Supplementary material for: Building integral projection models: a user's guide
Source: J Anim Ecol. 2014 Jan 20;83(3):528–45. doi: 10.1111/1365-2656.12178 (PMC4258094; doi:10.1111/1365-2656.12178)
Supplement: Appendix S4 — Constructing IPMs when more than one census is available. [file jane0083-0528-SD5.pdf]

Appendix S4: Constructing IPMs when more than one census is available

M. Rees, D.Z. Childs, and S.P. Ellner.
Building Integral Projection Models: a User's Guide.
Journal of Animal Ecology, 2014

In this section we outline a general approach for arriving at the "right" IPM in situations where more than one census (defined as any period of data collection) is performed each year. Using our Soay IPM as a case study, we show how this approach should be applied when one or more censuses are imperfect, in the sense that not every class of individual is measured. This is exactly the situation we face in the Soay system. Only lamb masses (measured shortly after birth) are acquired in the spring, whereas information about individuals of all ages are gathered in the late summer catch. Our aim is to show how to arrive at a model which is both *consistent* and *feasible*. By *consistent*, we mean a model that properly reflects the life cycle and census regime. By *feasible*, we mean a model that can also be parameterised from the available data.

The key to this methodology is to initially assume we have all the data we need at each census, and specify the corresponding model. We then "collapse" this model down in stages based upon our knowledge of which data are really available and our modelling objectives. In the Soay system, this means we first need to construct a model that projects the dynamics from late summer to spring (post reproduction), and then from spring to late summer again. To do this, we will need to keep track of both established individuals (denoted with a superscript E) and new lambs (denoted with a superscript L). The established individuals class includes every individual that survives to their first August catch. We distinguish winter and spring components of the demography with subscripts w and s, respectively. Spring size is denoted z^*. The remaining notation follows the conventions introduced in the main text unless otherwise stated.

As before, we aim to construct a model that projects the total August (summer) population size distribution, $n(z, t)$, between years. All individuals present at an August census are, by definition, established individuals. We begin with the equations for the spring size distributions of established individuals, $n^E(z^*, t + \tau)$, and new lambs, $n^L(z^*, t + \tau)$, produced by the August population at time t. These involve a winter survival-growth kernel, $P_w^E(z^*, z)$, and a winter fecundity kernel, $F_w^E(z^*, z)$, such that

$$\begin{aligned} n^E(z^*, t+\tau) &= \int_L^U P_w^E(z^*, z) n(z,t) dz \qquad & n^L(z^*, t+\tau) &= \int_L^U F_w^E(z^*, z) n(z,t) dz \\ P_w(z^*, z) &= s_w^E(z) G_w^E(z^*, z) & F_w(z^*, z) &= s_w^E(z) p_b(z) C_0^*(z^*, z)/2 \end{aligned} \tag{S4.1}$$

These expressions are very similar to the two components of the kernel from the Soay example, though everything in them now refers only to the winter transition. The fecundity component of the model does not contain a recruitment probability, $p_r(z)$, because this pertains to the next transition. We are also working with a different offspring size kernel, denoted C_0^* (not C_0), which describes the spring (not summer) mass of lambs.

The spring transition only involves survival and growth. The life cycle diagram tells us that the

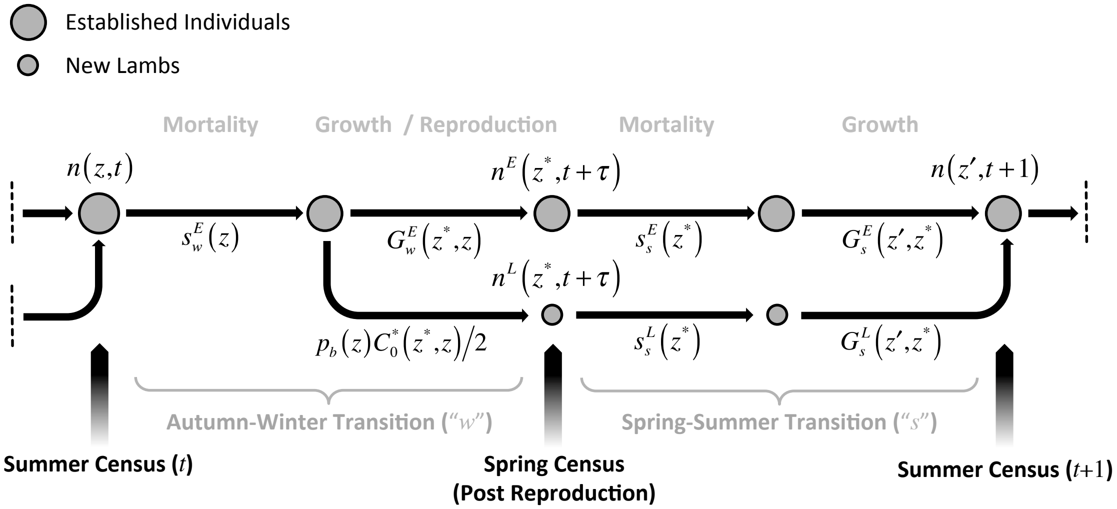

Figure S4.1: Expanded life cycle diagram for the Soay sheep. Two census points are shown: the summer census of size z individuals and a post reproduction spring census of size z^* individuals. The diagram shows the fate of established individuals, $n^E(\dots)$, and new lambs, $n^L(\dots)$ over the autumn-winter transition (w subscript) and the spring-summer transition (s subscript). The life cycle is conceptualised as occurring in 4 phases: (1) an autumn-winter mortality phase; (2) an autumn-winter growth and reproduction phase; (3) a spring-summer mortality phase; (4) a spring-summer growth phase. These processes are described by survival functions $s^{\circ}_{\circ}(\dots)$, growth kernels $G^{\circ}_{\circ}(\dots)$, a recruit size-distribution kernel $C^*_0(z^*, z)$ and a probability of reproduction function $p_b(z)$.

spring components of the model are

$$
\begin{aligned}
n^E(z', t+1) &= \int_L^U P^E_s(z', z^*) n^E(z^*, t+\tau) dz^* \qquad & n^L(z', t+1) &= \int_L^U P^L_s(z', z^*) n^L(z^*, t+\tau) dz^* \\
P^E_s(z', z^*) &= s^E_s(z^*) G^E_s(z', z^*) & P^L_s(z', z^*) &= s^L_s(z^*) G^L_s(z', z^*)
\end{aligned}
\tag{S4.2}
$$

The total August density function next year is then $n(z', t+1) = n^E(z', t+1) + n^L(z', t+1)$.

We now have a *consistent* model that properly accounts for the life cycle and known census times. This model projects from one summer to the next using two transitions: summer to spring and then spring to summer. It is therefore the IPM equivalent of a seasonal (or more generally, periodic) matrix projection model.

If we really had measured the size of all individuals in the spring, we could stop the model derivation here and begin to parameterise the various component functions from the data. However, in reality this is not a *feasible* model because only lambs were measured in the spring. There is no simple way to parameterise the established individuals' spring survival function, $s^E_s(z^*)$, and growth kernel, $G^E_s(z', z^*)$, in terms of spring size, z^*.

The solution to this problem is to collapse the survival-growth components of established individuals into a single transition. Instead of two survival-growth transitions governed by $P^E_w(z^*, z)$ and

$P_s^E(z', z^*)$, we have to model the summer-summer transition in a single step, such that

$$n^E(z', t+1) = \int_L^U P^E(z', z) n^E(z, t) dz$$
$$P^E(z', z) = s_w^E(z) s_s^E(z) G^E(z', z) \tag{S4.3}$$

In this new formulation we have combined the two growth phases into one summer to summer growth kernel, $G^E(z', z)$, that can be parameterised from the summer catch data. We are still separating the winter and summer survival processes, though now they must be expressed as functions of the preceding summer mass, z. Information about mortality is gathered over most of the year from regular visual censuses and by searching for dead individuals. Capture-mark-recapture methods could therefore potentially be used to estimate $s_w^E(z)$ and $s_s^E(z)$. However, this type of analysis is time-consuming and considerable expertise is needed to deal with missing individual size data (Langrock and King, 2013).

What else can be done to simplify the model? Some more knowledge of the system helps here. We know that virtually all the mortality of established individuals is experienced during the winter transition. This means that it is reasonable (and convenient) to set $s_s^E(z) = 1$. After combining equation (S4.3) and the lamb components of equations (S4.1) and (S4.2), following a little rearranging we get

$$n(z', t+1) = \int_L^U [P(z', z) + F(z', z)] n(z, t) dz$$
$$P(z', z) = s_w^E(z) G^E(z', z) \tag{S4.4}$$
$$F(z', z) = s_w^E(z) p_b(z) \int_L^U [s_s^L(z^*) G_s^L(z', z^*) C_0^*(z^*, z)/2] dz^*$$

Now let $s_s^L(z^*) = p_r$ (i.e. a constant), $s_w^E(z) = s(z)$, and $G^E(z', z) = G(z', z)$; and define $C_0(z', z) = \int G_s^L(z', z^*) C_0^*(z^*, z) dz^*$. This is essentially the same model we described in our example. The only difference is that here we have incorporated lamb spring-summer growth into the mathematical details of the model, whereas the in the original example we estimated $C_0(z', z)$ directly and allowed the data to effectively "do the integration" for us.

An important idea revealed by the derivation of the example model is that it will often be possible to construct a range of models of different complexity, particularly if more than one census is available. The choice of final model obviously depends upon the aims motivating its construction. If a model is being developed primarily to project the dynamics, e.g. to compare the population growth rates at different sites or explore transient dynamics, then it is reasonable to adopt the simplest possible model. This minimises the number of parameters we have to estimate and keeps the implementation simpler. If on the other hand the model is to be used to understand selection on the life history, e.g. via the calculation of parameter sensitivities, then it makes more sense to keep the components of lamb demography separated as in equations S4.4. This provides more insight into the effect of reproduction on population growth rate. The formulation we outlined assumes that there are no maternal effects on lambs that play out over spring and summer. This assumption could be relaxed, though it results in a much more complicated model because we have to keep track of the bivariate mother-offspring size distribution. However, this effort might be warranted if the model is going to be used to understand how phenotypic maternal effects impact the dynamics.

If we really had gathered size data for individuals of all ages at both censuses it would be natural to construct a seasonal IPM. Alternatively, we could ”collapse” the model to project the dynamics among years. We would then have to make a decision about which census point to use: spring to spring or summer to summer. The kernels would be different, but the resultant dynamics would be essentially the same. In both cases the resultant model would be of the post-reproductive census variety, because both reproduction kernels contain survival functions of the established individuals. What if we had collected spring size data prior to, instead of after, reproduction? In this case we could construct either a post-reproductive census model (by projecting from summer to summer) or a pre-reproductive census model (by projecting from spring to spring). This illustrates another feature of IPMs constructed from multiple censuses. The pre- versus post-reproductive census distinction is no longer necessarily a feature of the data collection methodology. Instead it reflects the life cycle, the data and our modelling decisions.

References

Langrock, R. and King, R. (2013). Maximum likelihood analysis of mark-recapture-recovery models in the presence of continuous covariates. *The Annals of Applied Statistics*, 7:1709–1732.
